# Baseline of visceral fat area and decreased body weight correlate with improved pulmonary function after Roux-en-Y Gastric Bypass in Chinese obese patients with BMI 28–35 kg/m^2^ and Type 2 diabetes: a 6-month follow-up

**DOI:** 10.1186/s12902-015-0027-0

**Published:** 2015-06-09

**Authors:** Yinfang Tu, Haoyong Yu, Yuqian Bao, Pin Zhang, Jianzhong Di, Xiaodong Han, Weiping Jia

**Affiliations:** Department of Endocrinology and Metabolism, Shanghai Jiao Tong University Affiliated Sixth People’s Hospital, Shanghai Diabetes Institute, Shanghai Clinical Center of Diabetes, Shanghai Key Laboratory of Diabetes Mellitus, Shanghai Key Clinical Center for Metabolic Disease, Yishan Road 600, Shanghai, China; Department of General surgery, Shanghai Jiao Tong University Affiliated Sixth People’s Hospital, Yishan Road 600, Shanghai, China

**Keywords:** Body weight, Obesity, Abdominal, Visceral fat, Diabetes, Type 2, Pulmonary function test

## Abstract

**Background:**

Associations between demographic data and pulmonary function have not been adequately examined in patients that underwent Roux-en-Y Gastric Bypass (RYGB). This study was designed to examine changes in body fat distribution and metabolic parameters after RYGB and whether these changes correlated with improved lung function.

**Methods:**

A retrospective review of 32 ethnic Chinese with obesity with body mass index (BMI) 28–35 kg/m^2^ and type 2 diabetes (T2DM) was conducted, focusing on metabolic outcomes and pulmonary function 6 months after RYGB.

**Results:**

Forced expiratory volume during first second (FEV1), percentage of forced expiratory volume during first second (FEV1 [%pred]), forced vital capacity (FVC), and percentage of forced vital capacity (FVC [%pred]) all improved significantly after RYGB. These increases all were negatively correlated with decreases in body weight and visceral fat area (VFA). The improvements of FEV1, FEV1 [%pred] and FVC were also negatively correlated with baseline of body weight and VFA. Furthermore, increases in FEV1 and FVC were independently associated with baseline of VFA (*β* = −0.003, *P* = 0.000; *β* = −0.004, *P* = 0.002, respectively).

**Conclusions:**

The baseline of VFA and weight loss induced by RYGB independently correlated with improved pulmonary function in Chinese patients.

## Background

Obesity adversely affects pulmonary function, especially in those exhibiting the restrictive pattern of functional impairment [1–3]. Such impairment is found in patients with impaired glucose tolerance (IGT), diabetes, metabolic syndrome and cardiovascular disease [3–5]. However, previous work examining the association of pulmonary function with abdominal obesity defined by body weight, waist circumference (WC), hip circumference, body mass index (BMI), waist-to-height or waist-to-hip ratio remains controversial [1, 6–9]. Studies using visceral fat area (VFA) to more accurately measure abdominal obesity in morbidly obese T2DM patients and examine the correlation with pulmonary function are lacking.

Roux-en-Y gastric bypass (RYGB) is currently performed in T2DM subjects with BMI over 35kg/m^2^, with evidence from the literature that RYGB may be appropriate for Chinese T2DM patients with BMI 25-35kg/m^2^  [10]. By criteria HbA1c <6.5 %, the pencentage of patients with diabetes remission were 61 to 93 % at 12 months after gastric bypass [11, 12]. For example, in T2DM patients with BMI < 35 kg/m^2^ who underwent RYGB, BMI decreased from 34.6 ± 0.8 kg/m^2^ to 25.8 ± 2.5 kg/m^2^ and HbA1c levels decreased from 8.2 ± 2.0 % to 6.1 ± 2.7 %, 1 year after RYGB [13]. Furthermore, the VFA: subcutaneous fat area (SFA), ratio fell substantially after surgical intervention in obese patients with T2DM, suggesting that weight loss after RYGB preferentially affects abdominal fat, particularly visceral fat [14]. Several studies have found that mortality during bariatric surgery is associated with respiratory complications [15, 16]. Postoperative respiratory complications including tachypnea, arterial oxygen desaturation, atelectasis, pneumonia, hypoxemia, and hypercapnia have all been noted [6]. Importantly, several studies that included long-term follow-up have found that weight loss after bariatric surgery greatly improves pulmonary function by increasing the forced vital capacity (FVC) [17, 18] and the forced expiratory volume in 1 s (FEV1) [18]. However, it has been proposed that ethnic differences exist among patients and, to date, neither cross-sectional nor longitudinal associations between demographic data and pulmonary function have been adequately examined in Chinese patients treated with RYGB. Moreover, no study has yet used the reduction in VFA measured via magnetic resonance imaging (MRI), an accurate assessment of abdominal obesity, to study the association with improvements in pulmonary function after RYGB.

In this study, we evaluated baseline and changes in obesity by using body weight, BMI, VFA, SFA and in lipid metabolism. We then examined their association with improvement of pulmonary function in Chinese obese patients with BMI 28–35 kg/m^2^ and T2DM, followed up to 6 months after RYGB.

## Methods

### Patients

This was a retrospective observational study approved by the Ethics Committee of the Shanghai Jiaotong Universtity affiliated No. 6 Hospital. Between February 2011 and January 2012, 32 Chinese patients (14 males and 18 females) aged 18 to 65 years, who were obese (BMI 28–35 kg/m^2^) and who had T2DM, underwent laparoscopic RYGB (LRYGB) in our hospital and were enrolled in the present study. All patients underwent identical anaesthetic and surgical protocols. All surgeries were performed laparoscopically using a standardized technique. A 25 mL gastric pouch was divided from the distal remnant. The biliopancreatic and alimentary limbs were 100–120 cm in length. Pulmonary function (spirometry), weight, BMI, VFA, SFA and lipid metabolism were evaluated in the preoperative period and 6 months after surgery. Smoking history was recorded. Any patient with a history of open abdominal surgery, a serious disease (such as a heart or lung insufficiency) that was incompatible with surgery, an acute T2DM complication, severe alcohol or drug dependency, a mental disorder, type 1 DM, secondary diabetes, an unstable psychiatric illness; who was a relatively high surgical risk (such as a patient with an active ulcer); or who was unable to adequately undergo pulmonary function testing was excluded. The written informed consent for participation in the study was obtained from participants.

### Pulmonary function tests

Evaluation of pulmonary function was carried out with each patient in a sitting position, with nose clips in place, via body plethysmography (Jaeger Masterscreen Body, Würzburg, Germany). All tests were performed by the same team of technicians, who followed the recommendations of the American Thoracic Society (ATS)/European Respiratory Society (ERS) [19]. Knudson’s reference equations were used to derive predicted values of pulmonary function test results. Each patient repeated each test at least three times (yielding at least two reproducible and acceptable maneuvers). The FVC, FEV1, and FEV1/FVC ratio were measured. The results were considered to be reproducible if the second-highest FVC and FEV1 values were within 5 % of the highest values. The highest measured FEV1 value and the corresponding FVC were coded to allow for computer analysis. The results were expressed in absolute values and as percentages of predicted normal values (FEV1 [%pred] and FVC [%pred]).

### Evaluation of VFA and SFA

VFA and SFA were measured using a Philips Achieva 3.0-T MRI system (Philips Medical Systems, Eindhoven, the Netherlands) fitted with standard array coils; all subjects were in the supine position. Breath-hold FISP images centred on the L4-L5 intervertebral disc were obtained using standard localizer images. The following parameters were employed: Repetition Time (TR) = 4 ms, Echo Time (TE) = 2 ms, number of slices = 12, slice thickness = 8 mm, image matrix 256 × 256, and field-of-view = 500 × 500 mm. The four slices that were best-aligned with the L4-L5 disc were analyzed with the aid of the SliceOmatic 5.0 software package (Escape Medical Viewer V3.2) to obtain VFA and SFA values by fitting spline curves to points on the border of the subcutaneous and visceral regions. Non-fat areas within the visceral region were also outlined via spline fitting and subtracted from total visceral areas [20] .

### Statistical analysis

All statistical analyses were performed using SPSS version 20.0 (SPSS, Chicago, IL). Data on normally distributed variables were expressed as means ± standard deviations and analysed with the aid of the Shapiro-Wilk test. Data on skewed variables were expressed as medians (with interquartile ranges). After verifying the normality of distribution, the paired *t*-test was used to compare preoperative and 6-month postoperative results. Pearson correlation analysis was used to search for relationships among weight, BMI, VFA, SFA, measures of lipid metabolism, FEV1, FEV1 (%pred), FVC, FVC (%pred), and FEV1/FVC. Stepwise multiple regression analysis was used to identify independent parameters correlated with changes in pulmonary function. All reported *P*-values are two-tailed, and *P* < 0.05 was considered to reflect statistical significance.

## Results

Patient characteristics, including age, gender, smoking status, BMI, weight, VFA, SFA and lipid metabolism data, before and 6 months after RYGB, are shown in Table 1. The average reduction in VFA after RYGB was 70.9 %. This was much greater than the average drop in SFA (44.0 %). Significant reductions 6 months after surgery were noted in weight, BMI, and levels of total cholesterol (TC), triglycerides (TG) and low-density lipoprotein cholesterol (LDL-c). The BMI returned to normal in 21 patients and, in 8, fell into the range of overweight (25–28 kg/m^2^); only 3 patients remained obese (≥28 kg/m^2^) despite significant reductions in their BMI.

**Table 1:** Demographic data, VFA, SFA and lipid metabolism of obesity and T2DM before and 6 months after RYGB

Variables	Before RYGB(V1)	After RYGB	Δ	*P*
n	32	—	—	—
Male/Female	14/18	—	—	—
Age (years)	45.09 ± 11.26	—	—	—
Smoker	7 (21.9 %)	—	—	—
Weight (kg)	84.2 ± 14.4	66.8 ± 11.0	17.5 ± 6.2	<0.0001
BMI (kg/m^2^)	30.7 ± 3.5	24.4 ± 2.4	6.37 ± 2.2	<0.0001
VFA (cm^2^)	123.5 ± 36.4	40.1 ± 25.0	87.6 ± 36.3	<0.0001
SFA (cm^2^)	278.9 ± 82.7	163.2 ± 71.9	122.8 ± 59.9	<0.0001
TC (mmol/L)	5.03 ± 1.07	4.27 ± 1.16	0.81 ± 0.91	<0.0001
TG (mmol/L)	1.69 (1.37 ~ 2.80)	0.98 (0.84 ~ 1.50)	0.74 (0.48 ~ 1.14)	<0.0001
HDL-c (mmol/L)	1.06 ± 0.25	1.17 ± 0.23	−0.14 ± 0.19	<0.0001
LDL-c (mmol/L)	2.86 ± 0.85	2.41 ± 0.88	0.55 ± 0.86	<0.0001

Data are mean ± SD or median (interquartile range). BMI: Body mass index, VFA: visceral fat area, SFA: subcutaneous fat area, TC: total cholesterol; TG: triglyceride; HDL-c: high density lipoprotein cholesterol; LDL-c: low density lipoprotein cholesterol

**Fig. 1:**
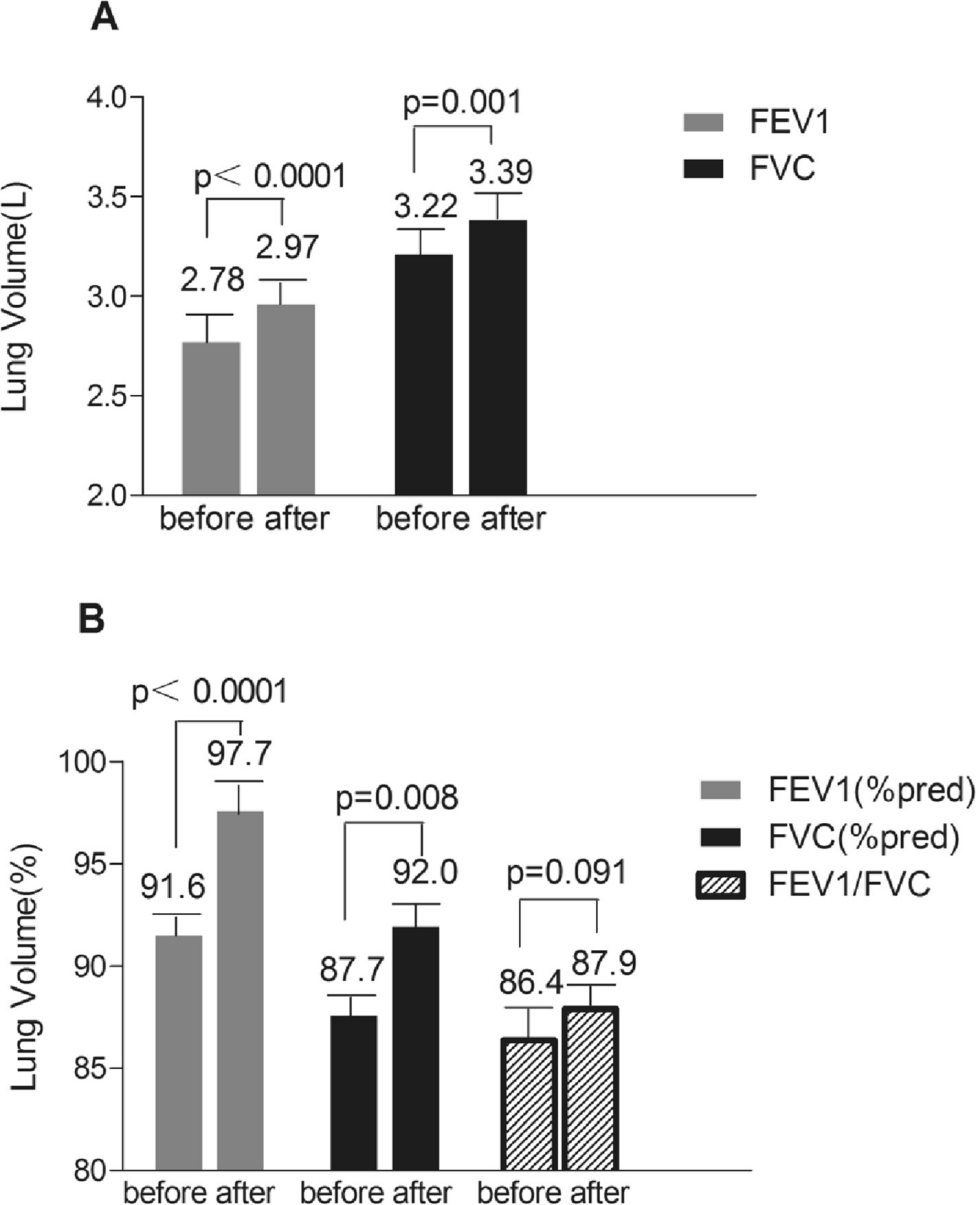
Pulmonary results of obesity and type 2 diabetes before and 6 months after RYGB. FEV1:forced expiratory volume during first second. FEV1(%pred): percentage of forced expiratory volume during first second. FVC: forced vital capacity. FVC(%pred): percentage of forced vital capacity. FEV1/FVC, %: percentage of ratio of forced expiratory volume in 1 s to forced vital capacity. Increase in **a** FEV1 and FVC. **b** FEV1(%pred), FVC(%pred) and FEV1/FVC. A paired *t*-test have been used here for statistical analysis

FEV1, FEV1 (%pred), FVC, and FVC (%pred) increased significantly after surgery. Although the FEV1/FVC ratio did not change significantly, a tendency toward an increase was evident (*P* < 0.1) (Fig. 1).

**Fig. 2:**
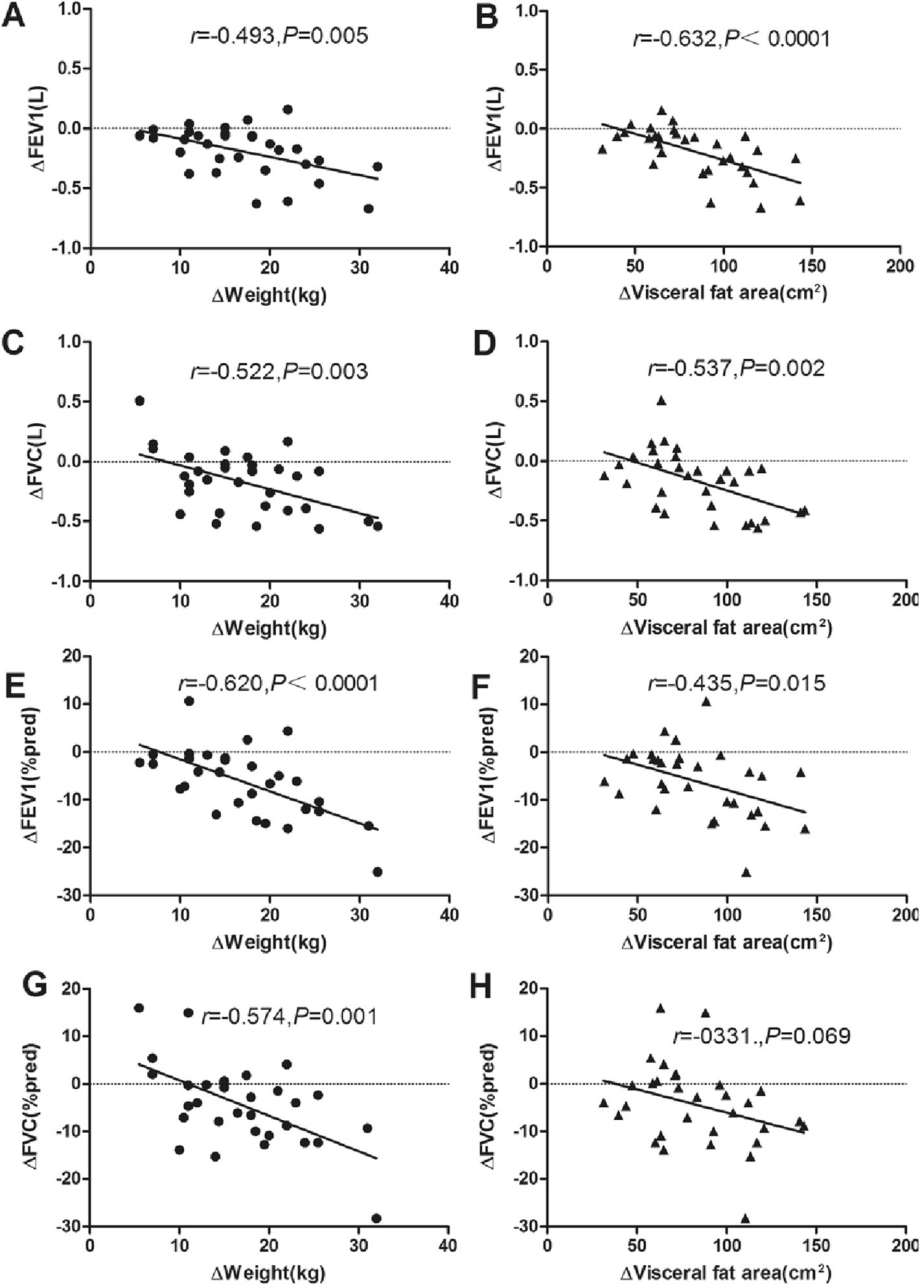
Correlations between the changes in weight and VFA and changes in FEV1, FEV1(%pred), FVC and FVC(%pred). Correlation of changes in **a** weight and FEV1. **b** VFA and FEV1. **c** weight and FVC. **d** VFA and FVC. **e** weight and FEV1(%pred). **f** VFA and FEV1(%pred). **g** weight and FVC(%pred). **h** VFA and FVC(%pred)

Increases in FEV1, FEV1(%pred), FVC and FVC(%pred) were negatively correlated with baseline and decreases in weight and VFA. Although increase in FVC(%pred) were not significantly correlated with baseline in weight and VFA, a possible correlation was evident (*P* < 0.1; Table 2 and Fig. 2). Furthermore, significant negative correlations were evident between increases in FEV1(%pred), FVC and FVC(%pred) and decreases in BMI, and between increases in FVC(%pred) and baseline of BMI. Increase in FEV1 was positively correlated with increase in HDL-c level. Significantly negative correlations were evident between increase in FEV1 (%pred) and decrease in TG level. Changes in pulmonary function were not significantly correlated with baseline of lipid metabolism (Table 2).

**Table 2:** Anthropometric and biochemical parameters showing significant correlations with changes in FEV1, FEV1(%pred), FVC, FVC (%pred)

	ΔFEV1		ΔFEV1 (%pred)		ΔFVC		ΔFVC (%pred)	
	Univariate^a^		Univariate^a^		Univariate^a^		Univariate^a^	
	*r*	*P*	*r*	*P*	*r*	*P*	*r*	*P*
ΔWeight (kg)	−0.493**	0.005	−0.620**	<0.0001	−0.522**	0.003	−0.574**	0.001
ΔBMI (kg/m^2^)	−0.354	0.051	−0.528**	0.002	−0.440*	0.013	−0.533**	0.002
ΔVFA (cm^2^)	−0.632**	<0.0001	−0.435*	0.015	−0.537**	0.002	−0.331	0.069
ΔSFA (cm^2^)	−0.039	0.836	−0.184	0.321	−0.046	0.808	−0.156	0.401
ΔTC (mmol/L)	0.217	0.242	0.115	0.539	0.058	0.757	−0.003	0.987
ΔTG (mmol/L)	−0.328	0.071	−0.208	0.261	−0.364*	0.044	−0.243	0.188
ΔHDL-c (mmol/L)	0.362*	0.045	0.116	0.535	0.069	0.711	0.123	0.510
ΔLDL-c (mmol/L)	0.306	0.094	0.165	0.374	0.236	0.202	−0.120	0.513
Weight (kg) V1	−0.462**	0.008	−0.398*	0.024	−0.381*	0.031	−0.320	0.074
BMI (kg/m^2^) V1	−0.280	0.121	−0.343	0.054	−0.333	0.062	−0.370*	0.037
VFA (cm^2^) V1	−0.642**	0.000	−0.439*	0.012	−0.534**	0.002	−0.336	0.060
SFA (cm^2^) V1	0.041	0.823	−0.029	0.876	0.021	0.909	−0.051	0.781
TC (mmol/L) V1	0.138	0.450	0.062	0.736	0.170	0.353	0.098	0.593
TG (mmol/L) V1	−0.191	0.295	−0.094	0.609	−0.183	0.315	−0.111	0.546
HDL-c (mmol/L) V1	0.342	0.055	0.096	0.601	0.278	0.124	0.067	0.717
LDL-c (mmol/L) V1	0.224	0.217	0.132	0.471	0.245	0.176	0.165	0.366

*P < 0.05, **P < 0.01

^a^Pearson’s correlation analyses were performed

Multiple stepwise regression analysis showed that increase in FEV1 (%pred) (*β* = −0.730, *P* < 0.0001) and FVC (%pred) (*β* = −0. 747, *P* = 0.001) were independently associated with decrease in weight, and that increases in FEV1 and FVC were independently associated with baseline of VFA (*β* = −0.003, *P* = 0.000; *β* = −0.004, *P* = 0.002, respectively), after adjustment for confounding factors. In addition, age was an independent risk factor for increase in FEV1 (%pred), and increase of HDL-c was an independent risk factor for increase in FEV1 (Table 3).

**Table 3:** Multiple stepwise regression analysis showing variables independently associated with changes in FEV1, FEV1(%pred), FVC and FVC(%pred)

	ΔFEV1		ΔFEV1 (%pred)		ΔFVC		ΔFVC (%pred)	
	Multivariate		Multivariate		Multivariate		Multivariate	
	*β*	*P*	*β*	*P*	*β*	*P*	*β*	*P*
Age (y)			−0.223	0.016				
ΔWeight (kg)			−0.730	<0.0001			−0.747	0.001
VFA (cm2)V1	−0.003	<0.0001			−0.004	0.002		
ΔHDL-c (mmol/L)	0.355	0.022						

A multiple stepwise regression analysis was performed. Age, gender, changes in weight, BMI, VFA, SFA, TC, TG, HDL-c and LDL-c, and baseline of weight, BMI, VFA and SFA were included in the original model

## Discussion

To the best of our knowledge, this is the first study to address the relationship between VFA and pulmonary function in obese T2DM Chinese patients. We observed that six months after RYGB surgery, significant reductions in BMI, body weight, VFA, SFA and TC, TG, LDL-c were evident, and HDL-c levels were significantly higher. Lung function improved markedly, based on FEV1, FEV1 (%pred), FVC, and FVC (%pred) values. This is in keeping with previous work which found that FVC and the FEV1/FVC ratio recovered in morbidly obese females 6 months after surgery, and that FEV1 recovered by 1 year [21]. Though the improvement was not so evident, it had significance. Because respiratory failure(blood gas analysis suggests type I or type II respiratory failure) was a contraindication for surgery, we enrolled patients with mild or moderate abnormal lung function, without apparent symptoms of dyspnea. We believed that studying a population with a higher BMI may suggest more benefit, especially if they were symptomatic. Considering the risks of anesthesia, we cannot yet enroll patients with apparent breathing difficulties.

Additional studies [6–8] have found that respiratory function improved with weight loss or waist-to-hip ratio, in morbidly obese patients 1 year after bariatric surgery. Paradoxically, waist-to-hip ratio did not significantly correlate with FVC and FEV1 after bariatric surgery in a Chinese population, although body weight, BMI, WC, hip circumference, and waist-to-height ratio did correlate well with FVC and FEV1 [1]. One study has observed that respiratory drive was reduced after weight loss induced by bariatric surgery [9]. In the present study, 6 months after surgery, we observed increases in FEV1, FEV1 (%pred) and FVC and clearly established that these were correlated with weight loss and baseline of weight. Others have reported similar results in populations varying in terms of ethnicity [1, 6–8].

Most importantly, we established for the first time that baseline and decreases in VFA was correlated with increases in FEV1, FEV1 (%pred) and FVC. In particular, baseline of VFA was independently and significantly related to increases in FEV1 and FVC. A reduction in the VFA may have improved respiratory mechanics, consequently increasing FEV1, FEV1 (%pred), and FVC, as shown by the significant correlations among these variables (Fig. 2). In several former studies, simple measurements of abdominal obesity such as WC, the waist-to-hip ratio, or the waist-to-height ratio were found to be correlated with respiratory function [1, 6–8]. Furthermore, an increasing number of studies suggests that WC may be a better predictor of pulmonary functional impairment than other anthropometric obesity indices [1, 2, 4]. In the present study, the average reduction in VFA after RYGB far exceeded that of SFA, and was associated with improved respiratory function 6 months later. By accurately measuring the extent of visceral fat via MRI in the present study, we have clearly confirmed that obesity, especially abdominal obesity, affects pulmonary function. We believe that direct measurement of VFA using MRI affords a more accurate assessment of obesity than does WC or calculation of waist-to-hip ratios. We appreciate that MRI use is impractical in large-scale observational studies, however there is no doubt that it confers more accuracy and diagnostic power when applied to correlating obesity with related complications.

Obesity may influence pulmonary function because excess fat accumulation in the abdominal cavity and on the chest wall affects chest mechanics, increasing the work of breathing, reducing lung volumes, rendering respiratory muscles dysfunctional, impairing gas exchange and reducing exercise tolerance [7, 17, 22–26]. Our results may be attributable to upward physical pressure on the diaphragm caused by the increased abdominal volume of obese individuals. There has also been great interest in understanding the role of altered lipid metabolism in contributing to altered pulmonary function after weight loss. In keeping with a previous study, [27] we found no association between LDL-c levels and pulmonary function. Moreover, we found that only HDL-c levels were independently positively correlated with FEV1 and this may reflect the role played by HDL-c in immunoregulation and attenuation of inflammation [28].

The present study has several limitations, including a small sample size and a limited follow-up duration, however the current data and conclusions provide important verification on the predictive accuracy of VFA measures. Although the small number of patients imposed limitations on analyses, we regressed our data to explore whether immediate postoperative respiratory functions were associated with gender, age, BMI, lipid metabolism, weight or VFA. In general, weight and VFA, but not SFA, influenced the rate of postoperative respiratory improvement. Another limitation is the lack of the control arm and hence it is not clear whether the findings are due to surgery itself or just the weight loss.

## Conclusions

We found that VFA decreased significantly 6 months after LRYGB. Decrease of VFA and weight loss after bariatric surgery were correlated to improved respiratory performance, as evidenced by the increases in lung volumes (FEV1, FEV1 [%pred], FVC, and FVC [%pred]) of obese Chinese patients with T2DM. The improvements in pulmonary function 6 months after LRYGB were independently associated with the extent of weight loss and, in particular, baseline of VFA. We propose that patients who have more visceral fat may obtain greater benefit from RYGB surgery than those with less visceral fat. However, further studies with higher sample size and long-term follow-up are needed to more conclusively establish the link between VFA and pulmonary functional improvement.

## Abbreviations

BMI: Body mass index
VFA: Visceral fat area
SFA: Subcutaneous fat area
TC: Total cholesterol
TG: Triglyceride
HDL-c: High density lipoprotein cholesterol
LDL-c: Low density lipoprotein cholesterol
FEV1: Forced expiratory volume during first second
FEV1(%pred): Percentage of forced expiratory volume during first second
FVC: Forced vital capacity
FVC(%pred): Percentage of forced vital capacity
FEV1/FVC: %, percentage of ratio of forced expiratory volume in 1 second to forced vital capacity
